# Recent advancements toward the incremsent of drug solubility using environmentally-friendly supercritical CO_2_: a machine learning perspective

**DOI:** 10.3389/fmed.2024.1467289

**Published:** 2024-09-02

**Authors:** Jawaher Abdullah Alamoudi

**Affiliations:** Department of Pharmaceutical Sciences, College of Pharmacy, Princess Nourah bint Abdulrahman University, Riyadh, Saudi Arabia

**Keywords:** supercritical CO_2_, solubility enhancement, machine learning, therapeutic efficiency, artificial intelligence

## Abstract

Inadequate bioavailability of therapeutic drugs, which is often the consequence of their unacceptable solubility and dissolution rates, is an indisputable operational challenge of pharmaceutical companies due to its detrimental effect on the therapeutic efficacy. Over the recent decades, application of supercritical fluids (SCFs) (mainly SCCO_2_) has attracted the attentions of many scientists as promising alternative of toxic and environmentally-hazardous organic solvents due to possessing positive advantages like low flammability, availability, high performance, eco-friendliness and safety/simplicity of operation. Nowadays, application of different machine learning (ML) as a versatile, robust and accurate approach for the prediction of different momentous parameters like solubility and bioavailability has been of great attentions due to the non-affordability and time-wasting nature of experimental investigations. The prominent goal of this article is to review the role of different ML-based tools for the prediction of solubility/bioavailability of drugs using SCCO_2_. Moreover, the importance of solubility factor in the pharmaceutical industry and different possible techniques for increasing the amount of this parameter in poorly-soluble drugs are comprehensively discussed. At the end, the efficiency of SCCO_2_ for improving the manufacturing process of drug nanocrystals is aimed to be discussed.

## Graphical abstract

Applications of supercritical CO_2_ in different industries

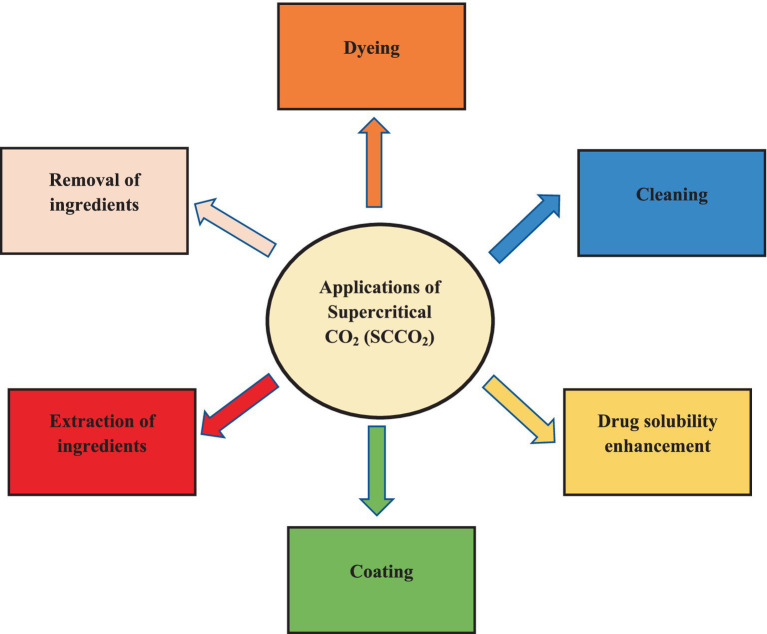

## 1 Introduction

Over the recent decades, the manufacturing process of innovative, affordable and effective therapeutic agents with optimum physicochemical and biological profiles has been the most important mission of scientists in the pharmaceutical industry (1–4). Despite great importance and significant endeavors, pharmaceutical companies have currently faced with different challenges like patent considerations, decrement in the number of novel marketed products, time/cost-intensiveness of R&D regulatory pathways toward the advancement of Active Pharmaceutical Ingredients (APIs) and their appropriate dosage of administration (5–9). It is momentous to note that some operational/functional parameters like bioavailability, solubility, dissolution rate, permeability and morphology can considerably influence the physicochemical property and therefore, the therapeutic efficacy of each determined drug (10–12). Nearly 65 to 75% of prevalent APIs are recently in solid structure or consists of solid APIs formulated as solid suspensions. Owing to the presence of important characteristics like simplicity of synthesis, higher physicochemical stability and crystallinity, formation of APIs in the solid state seems to be more appropriate. It is worth mentioning that safety, medical efficacy, physicochemical stabilities and sufficient solubility in aqueous media can be considered as momentous parameters for the development of novel API (13–16). In the current years, application of novel types of ionic liquids (ILs) in the pharmaceutical industry has paved the way for more efficient delivery of drugs. These novel, effective and green compounds have shown their great potential to deal with the challenges related to conventional dosage forms like insufficient solubility and low permeability in topical drug delivery systems (17, 18).

Drug formulation is usually implemented by the combination of inert excipients with active APIs for the production of highly-effective drug with favorable therapeutic efficacy. In the current years, serious endeavors are being made to improve/optimize the momentous parameters related to drug formation for decreasing side effects and enhancing API stability and patient compliance (19–22). On the basis of appropriate route of administration, formulation of APIs can be done via disparate set of materials like inert excipients (i.e., polymers, surfactants) and in an extensive type of delivery systems including microparticles (MPs) and nanoparticles (NPs) (7, 23–26). On-time innovation of highly-efficacious therapeutic agents to the market depends on the advancement of promising drug delivery systems. Despite the great success of conventional approaches for the formulation and discovery of effective drugs for patients, the emergence of several challenges like high cost and long process time has restricted their application in pharmacology (27–30). In doing so, finding novel, time-saving and affordable techniques for the prediction of drug properties is of great necessity.

**Figure 1:**
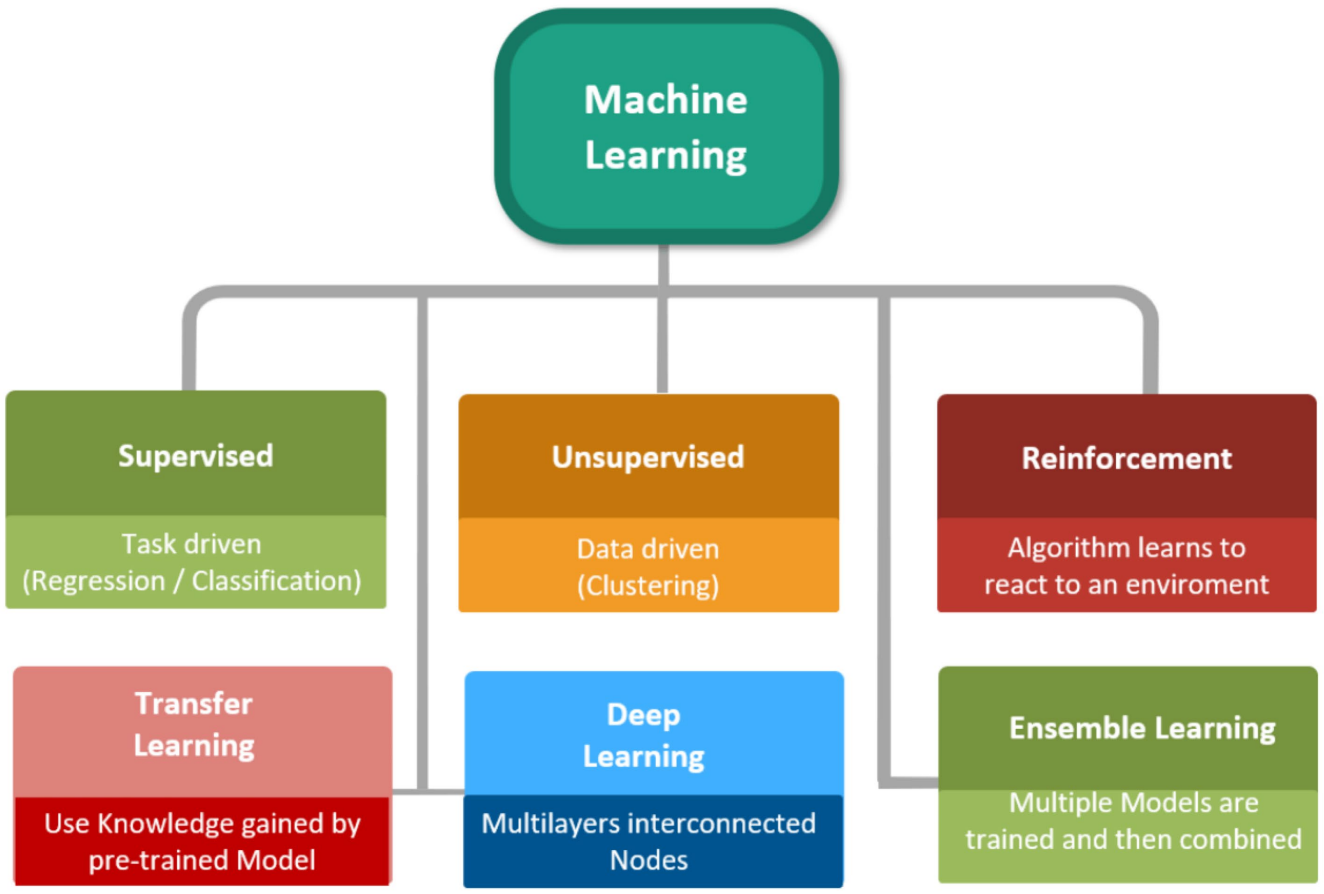
Various approaches of ML (106).

Machine learning (ML) can be interpreted as a subdivision of artificial intelligence (AI) that has illustrated its brilliant potential of utilization for the prediction of momentous parameters in different processes via training computational models on the basis of a body of data (25, 31, 32). Over the last years, artificial intelligence (AI) has substantially improved the formulation /development of novel therapeutic agents. Using AI, scientists are now able to optimize the formulation of drugs and increase the accuracy of clinical trials (33–35). For example, ML can facilitate the stability prediction of a special drug formulation by the consideration of data from numerous experimental investigations that evaluated the stability of API formulations. Current developments in ML algorithms as well as the advancement of robust and cost-effective have substantially enhanced availability to precise ML-based models (36–38). The abovementioned advancements have eventuated in the emergence of unbelievable tendency in the real-world utilization of different ML/AI-based algorithms in several industrial aspects like gas separation, cancer diagnosis, membrane processes, absorption/adsorption, catalysis and pharmaceuticals (39–45). Other noteworthy utilization of ML-based algorithms are the use of supervised learning algorithms and deep reinforcement learning for the estimation of momentous parameters in chemical reactions and the employment of deep learning (DL) to specify the 3D structure of a protein from its amino acid sequence (46–50). Figure 1 schematically demonstrates various approaches of ML.

This paper aims to review the role of disparate ML-based tools for the prediction of solubility/bioavailability of drugs using SCCO_2_. Moreover, the importance of solubility factor in the pharmaceutical industry and different possible techniques for increasing the amount of this parameter in poorly-soluble drugs are comprehensively discussed. At the end, the efficiency of SCCO_2_ for improving the manufacturing process of drug nanocrystals is aimed to be discussed.

## 2 Definition and importance of the solubility factor in pharmaceutical industry

**Figure 2:**
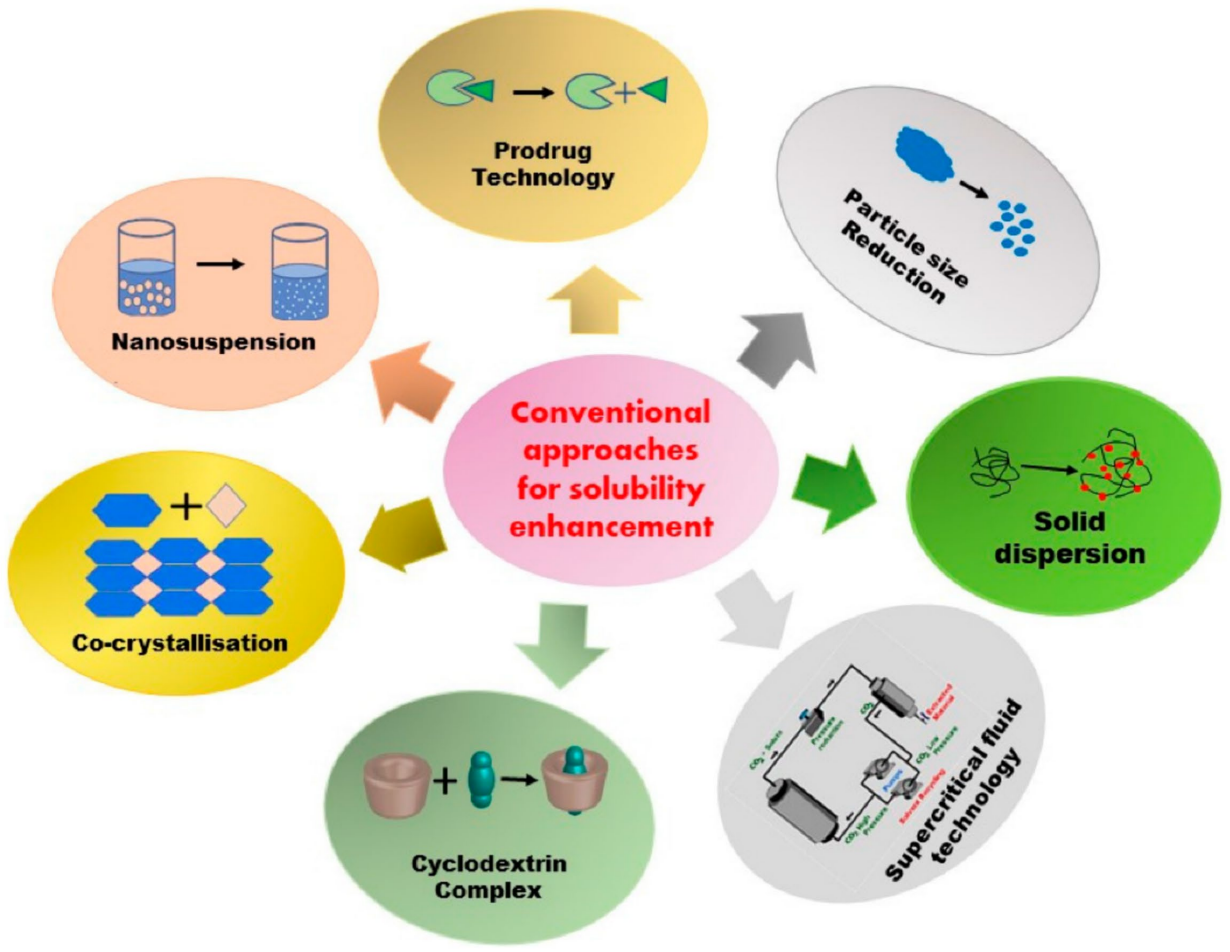
Schematic presentation of common techniques for enhancing the solubility of poorly-soluble drugs (57).

Solubility may be well considered as a momentous physical property and is defined as the ability of a specific solvent absorption in solvents. This factor is extensively employed in disparate scientific fields like liquid–liquid extraction, membrane-based separation, drug delivery, material science and medicine (51–56). Poor solubility of therapeutic medicines in water can be regarded as an important challenge in the pharmaceutical industry, which may lead to reducing the bioavailability and therapeutic performance. This challenge has motivated the researchers to develop novel ways to overcome this problem (56–58). Figure 2 schematically presents different common techniques for increasing the solubility of poorly-soluble drugs.

**Figure 3:**
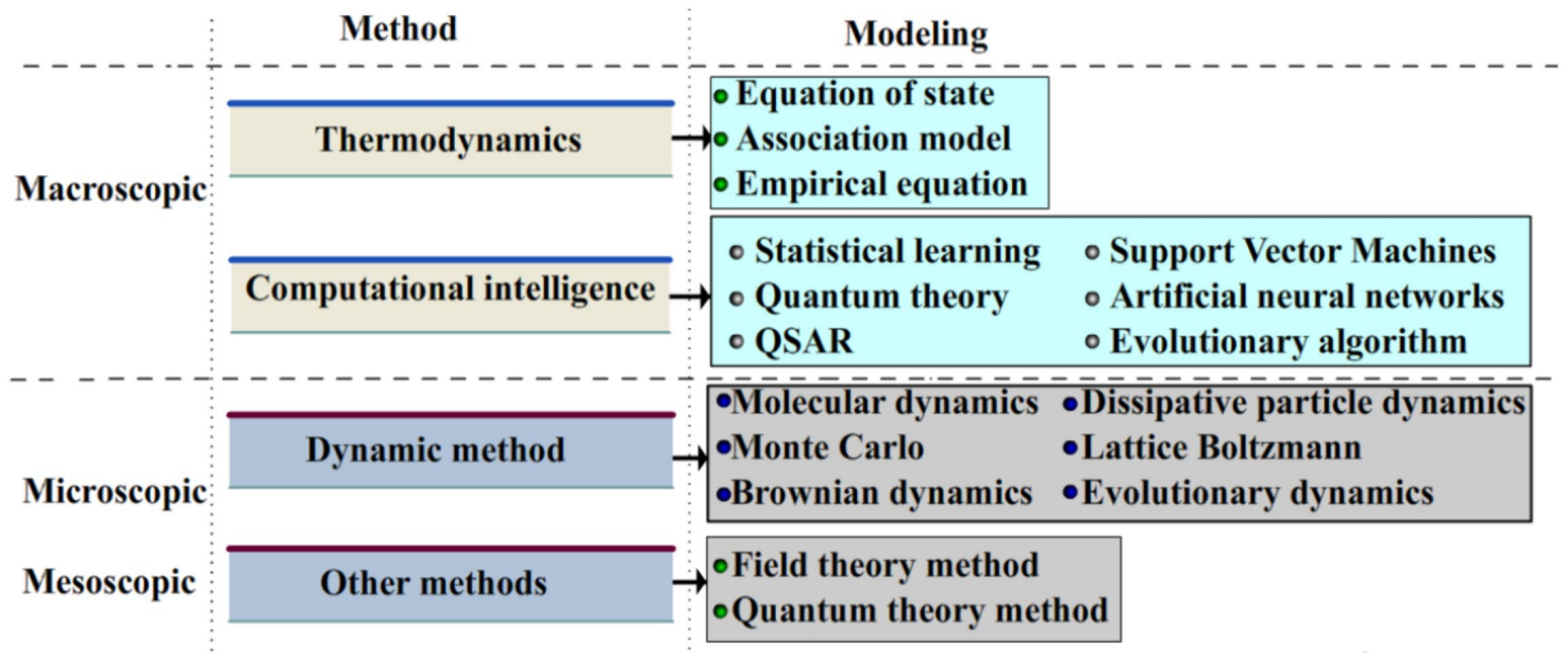
Schematic demonstration of employed models and methods of multiscale model. Reprinted from (54) with permission from Elsevier.

To conduct the scientific researches in the scientific scope of solubility, experimental analysis and computer simulation must be incorporated. Owing to the presence of various challenges during the conduction of investigational experiments under high temperature/pressure, particularly accompanying with stirring and vibration (with the aim of accelerating solubility), scientists have made their efforts to use accurate techniques for the prediction of momentous parameters related to drug development (59, 60). Over the last decades, development of predictive modeling tools has attracted the attentions of academic/industrial researchers. For instance, three eminent scientists (Martin Karplus, Michael Levitt, and Arieh Warshel) were the winner of the 2013 Nobel Prize in chemistry for the advancement of multiscale models for complicated chemical systems (61). The multiscale technique applies disparate time and space scales for the investigation of prevalent problem in all the micro-, meso- and microspatial scales (62–64). Figure 3 schematically demonstrates the employed models and methods of multiscale model.

It is of great importance to note the fact that the solubility of therapeutic drugs at the macroscale and its rate at the micro/meso scales results in the emergence of important challenges to the conventional research procedures (54, 65). The multiscale solubility investigation can be regarded as one of the most efficient procedures to show the inherence of dissolution process. This type of study has recently demonstrated great potential for the prepare scientific reference for process parameter selection at the multiscale level. This technique possesses great potential in various scientific scopes like self-assembly and dynamic evaluation of polymers (66, 67).

## 3 Employed ML tools for the development of the pharmaceutical formulation

The ongoing approach toward the development of novel drugs must be on the basis of the quality by design principles (QbD) (68–70). The primary level toward the implementation of this aspect is the interpretation of the quality target product profile (QTPP) and the critical quality attributes (CQA) of the product. By the identification of the relationships, the appointment of design space takes place, which provides an appropriate opportunity to optimize/control the product’s quality (71–75). From the past till now, quantitative evaluation and appointment of the design space were on the basis of experimental design, regression procedures and prevalent statistical analysis. ML algorithms [particularly Artificial neural network (ANN)], have been applied in different complex examples of QbD-based pharmaceutical development. Extensive application of ANN in pharmaceutical industry is prominently due to their brilliant characteristics like non-linear nature and great capability to make complex relationships between CMAs or CPPs with CQAs for disparate pharmaceutical dosage forms (76–81). For instance, Simões et al. (77) have assessed the role of using ANN predictive model based on ML approach for the QbD-based advancement of a poorly soluble therapeutic medicine fabricated in industrial settings and its comparison with the reference product and bioequivalence investigations. To implement this research, ANN was constructed using only 5 hidden nodes in 1 hidden layer. Development of this predictive model was on the basis of the use of hyperbolic tangent functions and its validation was corroborated by a random holdback of 33% of the dataset. Application of this model resulted in the emergence of valid prediction formulas for all 3 responses, with R^2^ values higher than 0.94 for training and validation datasets (78).

**Figure 4:**
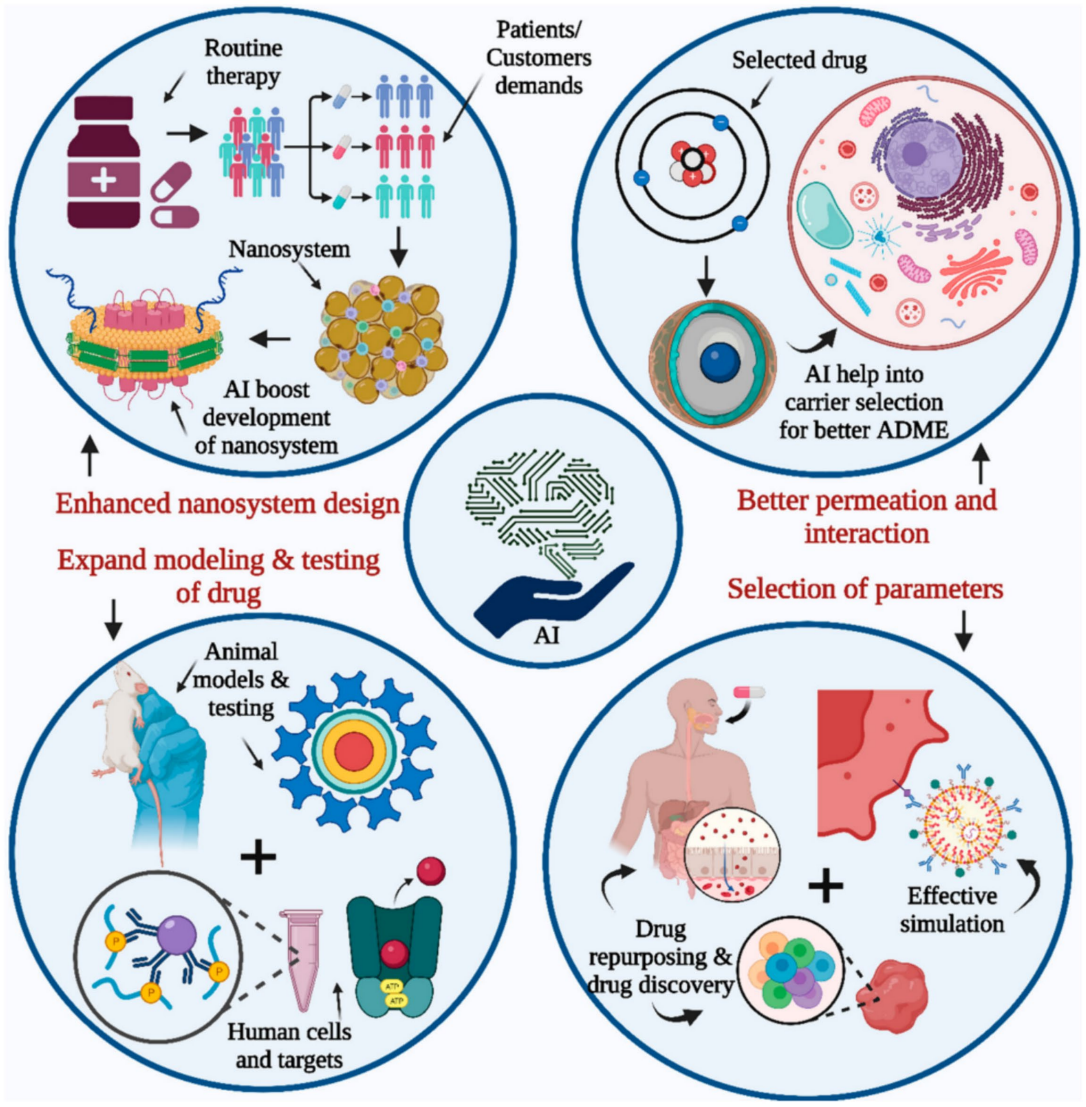
Schematic demonstration of AI contribution with drug development and research (88).

In a comprehensive scientific investigation, Belič et al. employed ANNs and fuzzy models to evaluate the impact of particle size and tableting parameters on the tablet capping tendency. The results demonstrated the fact that the developed model-based expert systems can dramatically enhance the trial-and-error procedures (82). Lee et al. developed an ML-based approach for the identification of emerging techniques at early stages applying immediately-defined multiple patent indicators. They also successfully applied a feed-forward multilayer neural network to provide the nonlinear connection between input and output indicators. They concluded that the developed approach could appropriately facilitate the responsive technology forecasting and planning in the pharmaceutical industry (83, 84). In the current years, the supremacy of deep learning (DL) than commonly-applied ML approaches has been recently approved (50, 85, 86). DL has illustrated their great potential of application in different industrial approaches such as the prediction of solubility and drug release (28, 87, 88). Apart from DL technique, other cutting-edge and breakthrough ML-based algorithms such as Light gradient boosting machine algorithm (lightGBM) have illustrated their noteworthy efficiency in the prediction of functional/operational parameters and complexation between cyclodextrins and APIs (89, 90). Decision tree (DT)-based techniques have shown their great potential to predict particle size of solid lipid nanoparticles (91). Figure 4 schematically depicts the incorporation potential of AI with drug development and research.

## 4 SCCO_2_ for increasing the solubility and manufacturing drug nanocrystals

The use of supercritical fluids for the formulation of drugs has recently attracted great attentions as an outstanding alternative for commonly-applied organic solvents owing to its brilliant positive points like non-toxicity, affordability and great efficiency (92–94). Rapid Expansion of Supercritical Solutions (RESS) is considered as the first technique of particle formation initially developed in the 1980’s (95). Despite the extensive application of disparate organic solvents (i.e., chlorodifluoromethane, trifluoromethane and ethane), A prevalent SCF in RESS is SCCO2 (96–98). Table 1 enlists the role of SCFs (especially CO_2_) solvents for the synthesis of the micro/nano amorphous/crystalline particles and micro/nano cocrystals using RESS method.

**Table 1:** Formation of micro/nano amorphous/crystalline particles and micro/nano cocrystals using RESS method.

API/bioactive substance	Employed SCF	Particle/cocrystal type	Particle size (nm)	References
Simvastatin	CHF_3_	Micro/nano amorphous/crystalline particle	47	(107)
Sirolimus	CO_2_	Micro/nano amorphous/crystalline particle	Less than 1,000	(108)
Raloxifene	CO_2_	Micro/nano amorphous/crystalline particle	18–137	(109)
Amoxicillin	CO_2_	Micro/nano amorphous/crystalline particle	1,080–5,720	(110)
Naproxen	CO_2_	Micro/nano amorphous/crystalline particle	560–820	(111)
Cholesterol-caffeine	CO_2_	Micro/nano cocrystal	81–169	(112)
Chlorpropamide-urea	CO_2_	Micro/nano cocrystal	N/A	(113)
Digitoxin	CO_2_	Micro/nano amorphous/crystalline particle	68–458	(114)
Felodipine	CO_2_	Micro/nano amorphous/crystalline particle	2000–6,000	(115)
Ibuprofen-nicotinamide	CO_2_	Micro/nano cocrystal	N/A	(116)

In the recent decades, pharmaceutical industry has encountered with different challenges. One of the most important concerns of scientists is the unacceptable solubility of therapeutic agents in water, which significantly affects their bioavailability, dissolution rates and hence, reduces the therapeutic performance of approved drugs (99). There are two prominent approaches that are being extensively applied in current years to overcome inadequate drug solubility concerns. (1) Techniques related to particle size reduction of drugs (i.e., micronization/nanonization). (2) Modification of physicochemical/structural characteristics of poorly water-soluble drugs (5, 100, 101). Currently, application of SCFs (particularly SCCO_2_), have been of paramount attentions to enhance the solubility/bioavailability and therefore, the therapeutic efficiency of medicines. Great interest toward the employment of SCCO_2_ is owing to its disparate advantageous technical properties, as well as noteworthy technical features like greenness, safety, versatility, simplicity of operation and low flammability (58, 102–104). Another practical CO_2_-based methodology for the formulation/synthesis of medical-based nanoparticles/nanocrystals or co-precipitated drugs is the application of SCCO_2_ as namely as high mobility solutes or co-solvents. By changing the use of this SCF from solvents to antisolvents and additives, the required value of CO_2_ to prepare micro/nanoparticles and the size of equipment to allow the needed CO_2_ action is declined dramatically (105).

## 5 Conclusion and future outlook

As mentioned in the manuscript, the integration of web innovation with medical science to enhance the precision and efficiency of predictive models in decision-making and deep learning algorithms. Thus, the motivation of scientists has recently been towards the use of different ML-based approaches in pharmaceutical industries. To reach this purpose, different ML-based approaches like support vector machine (SVM), multiple linear regression (MLR), radio frequency (RF) and deep learning techniques are being extensively implemented for the prediction of different momentous parameters like solubility and bioavailability. Over the last four decades, unique/tunable incorporation of brilliant and noteworthy physicochemical futures of SCCO2 with the growing regulatory needs and global requirements for more eco-friendly processes have increased the interest of researchers to develop and apply SCCO2-based bottom-up processes for enhancing the solubility and solubility of poorly-soluble therapeutic agents and forming drug-based nanoparticles/nanocrystals. This paper aimed to overview the role of different ML-based tools for the prediction of solubility/bioavailability of drugs using SCCO_2_. Moreover, the importance of solubility factor in the pharmaceutical industry and different possible techniques for increasing the amount of this parameter in poorly-soluble drugs are comprehensively discussed. At the end, the efficiency of SCCO_2_ for improving the manufacturing process of drug nanocrystals is aimed to be discussed. As the future perspective, different endeavors must be made to evaluate the rate of SCF-based reaction-mediated synthesis compared to conventional techniques. Moreover, the great potential of SCFs to manufacture environmentally-friendly and green extracts from plant substrates or industrial biowaste has motivated the researchers to pay more attention to the use of these solvents compared to organic solvents. Finding promising ways to facilitate the scale-up of these technologies can be another action, which can be under evaluation in the future for the production of pharmaceutical components, biowaste and different liquid extracts.
